# Journalists on COVID-19 Journalism: Communication Ecology of Pandemic Reporting

**DOI:** 10.1177/0002764221992813

**Published:** 2021-02-05

**Authors:** Mildred F. Perreault, Gregory P. Perreault

**Affiliations:** 1 East Tennessee State University, Johnson City, TN, USA; 2 Appalachian State University, Boone, NC, USA

**Keywords:** media ecology, metajournalistic discourse, coronavirus, crisis communication, COVID-19 communication ecology

## Abstract

In the midst of the COVID-19 pandemic, journalists have the challenging task of gathering and distributing accurate information. Journalists exist as a part of an ecology in which their work influences and is influenced by the environment that surrounds it. Using the framework of disaster communication ecology, this study explores the discursive construction of journalism during the COVID-19 crisis. To understand this process in the field of journalism, we unpacked discourses concerning the coronavirus pandemic collected from interviews with journalists during the pandemic and from the U.S. journalism trade press using the Discourses of Journalism Database. Through discourse analysis, we discovered that during COVID-19 journalists discursively placed themselves in a responsible but vulnerable position within the communication ecology—not solely as a result of the pandemic but also from environmental conditions that long preceded it. Journalists found their reporting difficult during the pandemic and sought to mitigate the forces challenging their work as they sought to reverse the flow of misinformation.

The normative role of a journalist is to share information of consequence with the public, and this is particularly important in the midst of a public health crisis like the COVID-19 pandemic. News organizations are normally part of a communication ecology, and so during the COVID-19 pandemic journalists and news organizations can be understood to be working in a *COVID-19 communication ecology.* Journalists serve as a resource for others within the ecology while also balancing personal challenges of the crisis.

According to a Wisconsin-based data journalist interviewed in November 2020 for this study, to be a journalist during COVID-19 is “to stump for the truth and to stump for critical thinking and to try to teach the importance of those things.” This journalist described having a personal responsibility to connect people with the resources they needed to stay healthy, particularly since at the beginning of the pandemic people “didn’t feel COVID-19 was a real threat—they felt that the media and government and healthcare is all conspiring to make this a bigger deal than it should be.”

Examining communication processes requires a comprehensive understanding of the environment in which communication exists. When sharing information about crisis and disaster, journalists exist as a part of an ecology in which journalism influences and is influenced by the environment. To understand communication ecologies, researchers often isolate certain practices and examine how they connect to other processes and data. The volatility of social media, fluctuation of information, and the desire to verify information about risks, health, and crisis provide a locus for dialogue about communication. In this way, communication ecologies provide a way to discuss broader issues. The boundaries of newer journalistic practices (data journalism, social media analysis, predictive journalism, and geolocated data) can prove challenging for traditional resource-strapped journalists in these circumstances.

How journalists talk about covering COVID-19 is informed by the larger conversation outside of the field of journalism. Metajournalistic discourse, or journalistic discourse about journalism, provides an avenue in which journalists assess their ecology and negotiate the boundaries of their work. This study explores how journalists discursively constructed their ecological relationships during the COVID-19 pandemic (with sources, their communities, information, and personal networks) as well as how they placed themselves within the ecology in relation to crisis information. We argue that journalists working during COVID-19 discursively placed themselves in a vulnerable position within the communication ecology, despite their responsibilities to facilitate relationships. Journalists hence found their reporting difficult during the pandemic, in that the pandemic exacerbated weaknesses that have long existed within the ecology.

## COVID-19 Communication Ecology

A disaster communication ecology (Spialek et al., 2016) refers to the resources and information individuals use before, during, and after a disaster. COVID-19 is considered a disaster in that it is “a potentially traumatic event that is collectively experienced, has an acute onset, and is time-delimited” (McFarlane & Norris, 2006, p. 4). While communication ecology is a broad-based approach to understanding how individuals communicate and gain information (Broad et al., 2013), communication resources used in a disaster and crisis situation may be different to those used outside of the disaster or crisis context. For example, tornado sirens or weather radios may not be regularly utilized outside of a tornado and therefore absent from an individual’s regular communication practices (M. F. Perreault et al., 2014). Similarly, journalistic relationships in a crisis or disaster might be similar to those outside of a crisis situation, but they might also be different given the circumstances around the crisis. For journalists working in COVID-19 conditions, interviewing people face-to-face might be difficult, but using online video conferencing software like Zoom could mitigate the challenge posed by pandemic restrictions.

Journalism exists as a microenvironment within the COVID-19 communication ecology. The dissemination of information by journalists relies on the capacity of journalists and the overall composition of the media environment. Therefore, the function of news in crisis and disaster communication reflects the overlapping of citizens, organizations, and journalists through social media (Houston et al., 2015). News organizations might engage the public online more often during a crisis or disaster, and therefore social media’s structure creates a much more complex ecosystem for information stakeholders (Paulussen & Harder, 2014; Weaver & Willnat, 2016). Hence, journalism serves as a conduit by which the general public may not only learn but also document and share information about a crisis.

While journalists, like any individuals, must adapt to crises and disasters, the COVID-19 communication ecology provides an environment where new norms and practices can be established and tried out, and perhaps innovated. This innovation is not new to the pandemic, but rather it results from journalists seeking to establish practices focused on four key principles: “research, a commitment to freedom of speech, a dedication to the pursuit of truth and accuracy in reporting, and ethics” (Pavlik, 2013, p. 181). From this lens, journalism situates itself in an environment not only of solid foundations but also of newfound fluctuation.

## Reporting in the Pandemic

In a research commentary released in the early stages of the pandemic, Lewis (2020) argued that the COVID-19 did not solely add new issues for journalists to work through but also compounded existing issues and enlarged “the blind spots in our work” (p. 683). In particular, Lewis (2020) explained, “Journalism research . . . tends to underplay some aspects of their lived experiences. Consider the complexity of covering crisis and trauma while also experiencing the same yourself” (p. 685). This applied to local journalists who are residents and stakeholders in the communities in which they work (M. F. Perreault, 2020). As Katz (1989) put it, newswork resembles the work of science. Journalists have a responsibility to be fact checkers and information relayers for the public. Journalists use formal, professional, informal, and personal means to contribute to the COVID-19 communication ecology.

In disaster and crisis communication, journalism serves as a conduit for communications from public officials and experts to the broader public—which is known as the facilitative role of journalism (Christians et al., 2010). Journalists conceive of and operate their role given what they perceive their audience needs. For example, research has shown that journalists operate in a storyteller role when mitigating coverage of dangerous actors (G. Perreault, Johnson, et al., 2020), an enrichment role when trying to comprehensively serve a particular community (G. Perreault & Bell, 2020), and a disseminator role when attempting to maintain objectivity in a volatile cultural space (G. Perreault, Stanfield, et al., 2020). In the facilitative role, the journalist’s purpose is to monitor, or observe, the environment for “relevant information about events, conditions, trends, and threats” (Christians et al., 2010, p. 139) and is operated in a response to a perceived need for collaboration. The facilitative role draws on an understanding that the journalist is responsible to society and perhaps contributes to the public’s decision making in a crisis.

## Metajournalistic Discourse

Metajournalistic discourse refers to the “site in which actors publicly engage in processes of establishing definitions, setting boundaries, and rendering judgments about journalism’s legitimacy” (Carlson, 2016, p. 350). As journalism’s institutional conversation, metajournalistic discourse primarily involves journalists talking about journalism (G. Perreault & Vos, 2020; Vos & G. Perreault, 2020). Some of this discourse is outward facing. The discourse is designed to be responsive and explanatory to the larger COVID-19 communication ecology (M. F. Perreault et al., 2014; Spialek et al., 2016). However, other discourse is inward facing and aimed to be discussed primarily by other journalists (Vos, 2016).

While conversations regarding what is considered to be a correct practice of journalism are discussed online through social media, they are also written about in columns and discussed in television programs that seek to understand why journalists are covering something a certain way and what the motivations are for covering it that way. The effort to assert professionalism and create more legitimacy becomes the primary concern for journalists arguing over what is deemed to be in line with consistent norms overtime (Carlson & Lewis, 2015). Crisis journalism provides a place for discourses regarding the changing journalistic paradigm (Berkowitz, 2000).

This discourse allows journalists to police their own field both formally—through public statements, letters from the editor—and, more commonly, informally—through discussion on journalism-oriented websites such as *Columbia Journalism Review*, *Poynter*, and *NiemanLab*. This policing occurs through marking actors and activities as either meeting institutional expectations or not meeting institutional expectations. Furthermore, this policing fills an institutional gap. Unlike other professions that operate with significant power, journalism lacks accrediting and licensing standards. Such standards would naturally run counter to journalistic autonomy, and thus, self-policing fills in the necessary institutional gap that allows journalism to police itself.

One critique of crisis journalistic practice is that in the past journalism has failed to see crises as an opportunity to assert itself as central to that crisis and open up new conversations and approaches (Zelizer, 2015). The nature of a crisis is to unseat norms. Any crisis presents a feeling of uncertainty, second guessing, and the urge to change the outcome—even as a challenge grows larger and becomes overwhelming (Bauman & Bordoni, 2014). Some editorial policies become more lenient in a crisis and in essence allow for conversations that would not normally occur, reflecting journalism’s layers of transformation in history (Carlson, 2016; Schudson, 1982; Stephens, 1989). However, in the pandemic, there is no benefit of hindsight given that there is no precedent for the COVID-19 environment. This leads us to pose the following questions:

**Research Question 1:** How do journalists discursively place themselves in the COVID-19 communication ecology in relation to coverage of the COVID-19 pandemic?
**Research Question 2:** How do journalists discursively construct their relationship with crisis information in relationship to the COVID-19 pandemic?

## Method

Given that this study is built on the framework of disaster communication ecology, we analyze the discourse most essential to journalists navigating that ecology. Hence, this study applies discourse analysis in order to place language in the context of institutional discourse (Fairclough, 2001) while circumscribing attention to “language in use” (Candlin, 2014, p. ix). In this case, the institutional context would largely reflect the seismic happening reflected by the COVID-19 pandemic. Such texts are a site of “struggle over meaning” in which a sort of “negotiation” occurs (Fürsich, 2009, p. 244). In short, journalism operates as a form of collective meaning making among actors that develops over time. As the crisis develops, so does the discourse, and, hence, this study will seek to place the discourse identified in its temporal context and the sequence of the discourse given that the sequence helps elucidate the implications of prior discursive positions (Carvalho, 2008). The need to control that discourse identifies specific goals that challenge journalism and its relationship to the current conditions of the world around it (Zelizer, 2015).

This study applies a two-step qualitative methodology. In the first step, interviews were conducted with eight journalists about their work during the COVID-19 pandemic. These journalists were identified via a purposive sampling method. The first six interviews were conducted in the early phases of the pandemic (March to April 2020). An additional two interviews were conducted in November 2020 to reflect on the earlier gathered information. In the second step, discourse regarding the pandemic was collected from the U.S. journalism trade press using a custom searchable database, the Discourses of Journalism Database. The trade press database was created by using a web scraper to download and archive articles and posts from 25 sites where journalists discuss journalism. This includes sites such as *Editor & Publisher*, *MediaShift*, *NiemanLab*, and the *Columbia Journalism Review*. The purpose of the database was more to choose sites that represented institutional discourse and provide less attention to sites at goal. The database compiled 83 articles that matched the keyword search *coronavirus* and that dealt specifically with journalistic discourse related to the pandemic.

Interviews were conducted throughout the research process but started prior to the collection of articles. An initial set of interviews with journalists was conducted at the beginning of the pandemic in March and April 2020—the interview transcripts were analyzed to provide a more comprehensive picture of the disaster ecology. A final set of interviews was conducted in November to reflect on the relationship indicated in the prior interviews and by the metajournalistic discourse. Because the pandemic was a global crisis when these articles were collected, the study takes place in medias res of the crisis cycle with resolution looming months in the future of the writing of this study.

Analysis of the discourse involved both authors reading the transcripts and articles for discursive themes, with particular awareness paid to the temporal context, how journalists identified institutional relationships, and how journalists navigated providing public information. Each of the coauthors analyzed the entirety of the sample with the research questions in mind and then compared themes to address the questions. In addition to those findings, the researchers also conducted individual interviews with local journalists that helped to contextualize this information. That said, we will provide relevant context for each discursive element to more clearly indicate both the development of the discourse and the crisis phases within the ecology.

## Results

### Vulnerability in COVID-19 Journalism

With regard to Research Question 1, journalists discursively articulated themselves in a vulnerable position in the COVID-19 communication ecology. Journalists saw the pandemic as laying bare the endangered nature of journalism, which was a result of pressure from access to sources as well as market forces. This jeopardized journalists’ ability to fulfill their responsibility to society.

In interviews, the journalists described their responsibility to share information that might help readers save their lives and the lives of others. For one Tennessee-based journalist, the challenges of reporting in a pandemic centered on providing adequate coverage for the community despite a lack of prior experience of reporting in a crisis. The journalist talked about the personal concerns he had for the community and how those concerns affect his reporting of the issues:

Before this outbreak, I’d had little to no knowledge or experience in crisis communication or how to respond to a crisis personally. . . . And while I was, in every sense of the phrase, thrown into the fire I managed to do what I could and work through it. (Since March 2020) the tone of the stories we’re writing has shifted to focus on the future: How will restaurants reopen? How will salons and gyms? How do we stay safe as we go out more? Is the worst over? Breaking news has come every week, and almost every day, whether that’s new case numbers, new developments in what we’re seeing or changes in policy. But we’ve also been feeding in concrete details and analysis with almost everything we’re doing.

This journalist perceived his risk of contracting COVID-19 to be the same as that of many readers and felt it was important to take his vulnerability seriously and communicate that to readers. This journalist’s office was closed, so he was reporting mainly from his home using a laptop and calling sources he had established before the pandemic lockdown. He periodically left his home to attend press conferences at social distance for the regional hospitals. His newsroom had already been reduced to five full-time reporters prior the start of the pandemic. For this journalist, the very action of reporting on the crisis helped him see the issues that already existed in his newsroom. He saw his work as a journalist as helping the public cope. This is the type of emotional labor that often gets overlooked as a result of journalists working on deadlines and attempting to be objective in their process (Lewis, 2020; Wahl-Jorgensen, 2020).

In the metajournalistic discourse, journalists indicated that they saw the pandemic as revealing—and at times accelerating—the already endangered nature of their profession. From the perspective of journalists, newsrooms were already facing challenges before COVID-19. In other words, “It’s clear that the coronavirus didn’t start the industry garbage fire as much as it threw accelerant on it” (Allisop, 2020).

Largely, journalists saw community newspapers as suffering the most substantially from the *accelerant* of the epidemic. Local broadcast newsrooms were seen as suffering nearly as much as their in-studio operations were reduced to comply with COVID-19 restrictions (Matloff, 2020). The least affected newsrooms were newsrooms already prepared to “go mobile on short notice” (Matloff, 2020). Matloff (2020) argues that California newsrooms were uniquely prepared, given that they have a “leg up when it comes to operating in the midst of an emergency.” This is not only a result of newsrooms operating amid a “history of catastrophic earthquakes” but also because they worked in a culture that anticipated challenges to their reporting norms.

At the *San Francisco Chronicle* in 2019 the newsroom used the anniversary of the earthquake of 1906 to conduct a work-from-home drill, having the entire staff work remotely. Later that year they conducted another drill on the anniversary of the 1989 Loma Prieta earthquake. By the time COVID-19 hit, the newsroom already knew how to work remotely. What the *Chronicle* has adapted to, according to Trewinnard (2020), was the need for distributed teams. Trewinnard argues that this team-oriented approach is the key to economic and physical survival, given that “a very practical benefit of distributed teams is saving the cost of maintaining a physical office.” This adaptation is not a “‘nice to have’—it’s an essential, foundational ingredient of a commercially sustainable newsroom operating in a low-trust, noisy information ecosystem awash with disinformation and misleading claims” (Trewinnard, 2020). Hence, journalists not only identified their vulnerability as longer standing than the virus but also identified the needed method of adaptation.

Adaptation also spilled into newsrooms where journalists began to cover COVID-19 in all aspects of their work, as every story became a COVID-19 story, according to a Tennessee-based journalist:

The biggest thing I’ve learned from covering the pandemic is just how important it is to be versatile as a reporter. . . . We’ve seen sports writers move to various beats when sports were canceled at major newspapers, and we’ve seen virtually every reporter covering stories they normally wouldn’t if it wasn’t for the pandemic. I tend to write a lot of feature and enterprise stories at the Press, and while I still do that, I’ve also stepped into breaking news, healthcare and education more than I normally would. The pandemic has really tested me in that regard, and while I feel I’ve handled it as well as can be expected, it still reinforces the importance of being a versatile reporter.

While the ability to interact with individuals in person was removed, there was an increased capacity for reporting, given that travel expectations were removed. Many journalists’ prepandemic sources were distributing official statements and holding virtual press events. Journalists spent more time researching stories from home and combing social media sites for leads. While the process of reporting and writing was more adaptable, there was not a guarantee of job security, and so there was an expectation to do more than they had done before the pandemic and to do it well.

Journalists felt pressure as a result of the economic consequences of market pressure. For example, community newspapers faced “economic consequences” such as the need to “reduce staff” or “close entirely” (Eisner, 2020). As proof of point, Eisner (2020) pointed toward the case of Britain’s *Jewish Chronicle*: “Already struggling with sinking revenues and shrinking readership, the Chronicle had succumbed to the human and financial wreckage caused by the global pandemic.” Journalists noted that the financial crisis revealed by coronavirus affected far more newsrooms than just a few individual cases. In March and April 2020, numerous newsrooms announced a variety of responses to the pandemic that included not only layoffs but also furloughs and salary reductions. And while larger media companies argued that they were “storm-weathering measures,” journalists were skeptical of such claims (Allisop, 2020). From their perspective, why would media companies waste a good crisis? “Amid the devastation, we must remember the industry’s underlying health conditions; asserting that the ‘coronavirus is killing our industry’ risks obscuring the deeper reasons for the malaise, and letting those responsible for it off the hook” (Allisop, 2020).

Hence, while journalists had some idea of what was needed to adapt, they also felt a need to hold the forces placing them in danger to account.

Journalists were not solely self-serving in articulating a desire for their own survival—rather, they felt that their mission of public service was in jeopardy. At times, journalists articulated this specifically to their given audience, as in Benton (2020b): “We feel a responsibility to the 25 percent of Washingtonians who lack broadband access, and the 17 percent who lack access to a computer.” At other times, the audience was the public at large—as the editor-in-chief argued in a question-and-answer piece—“our audience is all over the country and across partisan lines. I felt we had an opportunity and responsibility to provide information. It was an important first step for us” (Welton, 2020). And while it would certainly be understandable for journalists to be concerned with their own survival for their own sake—and they certainly articulated that too—they privileged the effects of their vulnerability in the ecosystem when discoursing the pandemic.

### Journalists Filtering Misinformation

With regard to Research Question 2, journalists constructed their relationship with information during the pandemic as innately problematic. The challenges of the pandemic, the proliferation of misinformation, and the need to carefully sift through the information required the same discernment applied to other forms of crisis. Working to prove the validity of certain information could be trusted was sometimes more difficult for journalists. In the study’s later interviews, one journalist indicated that people no longer trusted sources they had trusted in the past:

A key part of that is just understanding the distrust that a large percentage of our readers are going to have certain sources, with fact checking in particular. A year ago, we could say the (Centers for Disease Control) says this, and that was the end of it. Now we’ve got a country that has been conditioned in an echo chamber into believing that the CDC is not a reliable source of disease information.

His job working for a Wisconsin-based paper, and providing fact checks of information, took on new life beyond his job as he often posted his research on his Facebook page and debated with others about legitimacy of misinformation. He said this was somewhat more challenging because of the interaction between the pandemic and the 2020 U.S. election.

In early April, Mia Malan of the Bhekisisa Centre for Health Journalism in South Africa noted that journalists had a responsibility to follow up on rumors. Malan’s news organization had decided to “not cover all rumours because you can cross that line where you actually make people more aware of them, and they start to believe it. So, we’re very selective about that. It’s not even 10% of our coronavirus coverage” (Gupta & Flueckiger, 2020). This decision makes sense given Trewinnard’s (2020) description of the ecosystem as “awash with disinformation and misleading claims.” Journalists credibility is low according to Nelson (2020). This confusion is in part a result of competition with misinformation. Nelson argues that misinformation campaigns “routinely flood social media platforms and run the risk of conflating real news with fake news in the minds of the public.” Journalists indicated a reliance on health care experts to try to address the very complicated questions posed by their audiences. Questions such as “Hey, if I get infected, how likely is it that I’m going to die?” require journalists to process “through which scientists or researchers or doctors have gotten” in order to answer the question with the best information available (Harris, 2020). The problem however is that while audiences want this question answered, they do not necessarily trust the answers provided by journalists. Nelson (2020) notes that “a recent survey found that journalists were the least trusted spokespeople about the virus. People in 10 countries expected more truth-telling from health care CEOs . . . than from journalists.” This reflects not only the endangered state of journalists—as discussed earlier—but also the degree of competition journalists perceived with health care leaders.

Worth noting is that reporting collaboratively with medical experts was a taken-for-granted expectation in this sample. By contrast, journalists at times focused on the dangers of placing trust in the health care industry. The *Los Angeles Times* released an eight-part coronavirus video series (https://www.latimes.com/science/science-behind-coronavirus-123) starring transplant surgeon and owner of the *Los Angeles Times*, Patrick Soon-Shiong (2020). Soon-Shiong is also a medical researcher and owner of numerous medical companies. While Benton (2020a) admits that you probably “want his opinion on COVID-19 ahead of, say, A. G. Sulzberger or Sam Zell’s,” he also noted that the *Times* “may have reached a new high in combining its editorial products with its owner’s side gig.”

All of this together indicates that while journalists placed a high value in working with medical experts, they saw this collaborative work as facing multiple dangers: dangers of misinformation, dangers of having their work ignored as a result of their perceived lack of credibility, and, relatedly, dangers of having their work perceived as biased.

## Discussion

In a commentary, Lewis (2020) calls on journalism scholars to ponder the COVID-19 pandemic as a time to consider that “the news media landscape on the other side of the pandemic may look very different (and vastly diminished) from the one we knew before” (p. 682), and hence, researchers should consider “under-emphasized certain dimensions of journalism” (p. 683). The present study seeks to do that by examining how journalists navigate the relationships embedded in the COVID-19 communication ecology.

The crisis looming in journalism is one that was drastically augmented by the COVID-19 pandemic. Understanding journalists as facilitators for scientific information is not new but became central to the discourse in journalism during the COVID-19 pandemic. The importance of journalists conducting their own message assessment and meaning making helps them focus on coping as well as covering the crisis (Veil et al., 2008). Journalists want to get and provide information that is creditable and timely. The problem with the crisis of COVID-19 is that it eliminates the ability to segment crisis experience from the work of reporting on a crisis. Local information if often important to journalists, but due to COVID-19 journalists’ ability to interview people in person was often limited. A number of metajournalistic pieces emphasize the importance of focusing on local news organizations, many which have been gutted as a result of consolidation and created significant news deserts.

In order to connect readers with sources that will establish patterns of accuracy through transparent business practices, journalists often disclose their processes of verification to the public. While journalists have a long-standing concern for the process of verification, during the early phases of the COVID-19 crisis, the metajournalistic discourse emphasized concerns regarding credibility and news validity in discussing journalistic work with other entities. By mid-April 2020, the metajournalism focused on mitigating the already problematic information ecosystem while still tolerating a degree of uncertainty. Collaboration and transparency were referenced throughout the articles—with most of the concerns about factual information and long-term crisis appearing later in the sample. The metajournalism collected, when viewed temporarly, emphasized a critique of news practices and economic constraints as well as pandemic opportunities near the end of the sample. Changing perspective, even if it is because of the pandemic, can change the orientation of journalism from saving large buildings with a significant number of empty desks to focusing on topics directly affecting the community these journalists live in.

Journalists seek to legitimize certain reporting practices and establish consistent norms, not only to build trust but also to contribute to the public outlook and conversation (Carlson & Lewis, 2015). Often journalists are strategic in the ways in which they approach a topic out of concern for its future interpretation, or how it might be affected by unknown facts or information. In the case of COVID-19, journalists had to be vigilant to these changes. However, journalists at the same time sought to hold accountable those who are putting them and those they care for at risk.

Journalists discursively placed themselves in a vulnerable position as a result of the economic consequences of market pressure, given that many were experiencing the effects of the pandemic in their own communities, families, and careers. The idea that journalists are themselves able to facilitate these conversations with the general public while also serving as a conduit for verification becomes problematic, in that what is attempted is not always what is enacted, given the access to information, abilities, capacities, and desires of journalists themselves. The pandemic presents a gap between journalistic understanding and journalism practice.

Our results indicate that journalists constructed their relationship with information sources during the pandemic as innately problematic given the circulation of misinformation. AsKatz (1989) described, the challenges of doing journalism well in times of crisis is that the ecology may present more challenges than revelations in its established form. This means that journalists and journalism represent a number of the concerns for the public—including those for accurate information and long-term concerns like economic stability of the news and media industry. Pandemic pressures are augmented by the pressures to not only get the correct information out to the public but also overcome the challenges of reporting in these circumstances where stress is high and people are concerned about the long-term effects of the disease. Even in June 2020, when many journalists were returning to work (after many states lifted quarantine orders), many health agencies were not releasing consistent messaging about social distancing, mask wearing, susceptibility to the disease, and long-term effects because the information was still very new. Journalists remind each other that they must be scientific in their process, just as the scientists and doctors are with the disease—this metaphor speaks to the idea that journalists serve a vital function in crisis situations by relaying information to the public in an understandable manner and with clarity.

A recent trend toward journalists engaging actively with their audiences through online platforms has yielded more discussion about emotional labor as a central component of contemporary journalism. Research addressing audience engagement indicates that emotionally engaged audiences are more likely to “recall information and take action when news stories are relatable” (Wahl-Jorgensen, 2020, p. 175). Pandemic reporting plays a role in both institutional and public discourse in an event like COVID-19. The facilitative role not only creates a tension with the journalists’ individualism but also reflects their desire to compete with an overabundance of information posted by numerous sources. Performing this role in a pandemic requires an understanding that certain traditionally established norms or practices take a backseat to facilitating access and providing verification of information of consequence.

Journalists’ attempt to be proactive and innovative appears to be a way of coping with this lack of consistent and clear information. However, their flexibility and innovation is overshadowed by the limited resources journalists already had prepandemic. Perhaps the pandemic conditions only confirm that these hurdles are real and have been destroying the process of journalism in a number of ways.

### Limitations and Future Research

This study is dependent on its sample, which is bounded by time and is situated by the informal conversations around the roles of journalists through metajournalistic discourse. Future research would benefit from examining the political and societal engagement of journalists as well as rooting the ecological findings of this study in the perceived influence of the ecology on their actual reporting. These data also allude to market challenges and constraints that existed pre-COVID-19, which directly affected the way in which the pandemic was reported. Considering the pandemic as a unique crisis might illicit more insights into the adaptability of journalists during smaller and more time-delineated compounding crises like natural disasters. At this point, the pandemic has changed many professions if only as a result of the growing dynamic of remote work, online research, and access to information.

Another take away of this study is the role of metajournalistic discourse within the media ecology—which in this case demonstrated the potential of such discourse to help shape relational connections in the midst of a crisis. Another way journalism is held accountable is through the marketplace, be that economic or a marketplace mentality of discourse as expressed where journalism contributes to the public sphere (Garman, 2019). For instance, the Poynter Institute provided journalistic best practices in COVID-19, which included an interview with Erin Ailworth, a veteran *Wall Street Journal* reporter, regarding her COVID-19 reporting (Grau, 2020). Ailworth argued,

You have to think outside the box, especially with this pandemic, where we’re being asked to employ social distance, self-isolate and quarantine. . . . Opening a window into the reporting process, I think, is more important now when we’re all so physically disconnected. (Grau, 2020).

The interviews with journalists for this study discussed these challenges, as augmented pressures to provide legitimate information in order to literally save lives in the general public. The ability to adapt was emphasized as essential by the journalists interviewed and in the metajounalistic discourse. With all the risks associated with reporting in a pandemic, adaption was seen as vital to not only coping but also getting work done, and maintaining safety. Journalism presents a conversation on the context for journalistic work. Context refers to the environment in which the medium is used—and how media affect society and demonstrate certain professional norms. The study not only brings to the forefront issues that already existed in journalistic practice but also provides a focal point for those conversations in relation to journalism’s purpose in the pandemic environment and greater society.

## Conclusion

There are several conversations that require further investigation and attention moving forward. The pandemic is still in process and journalists are still dealing with the daily challenges of the crisis. At the time of writing, there is not an end in sight to the pandemic, despite the early arrival of vaccines, and certainly no end in sight for the effects of the pandemic on the media industry, journalism, and journalists. The COVID-19 communication ecology provides a place to dissect the ways in which journalists do their work and also think about their work; perhaps the journalism industry will become more flexible or change to adapt to the current and future ecology. The disaster communication ecology requires journalists to maintain consistency and adapt to the changing expectations of the public while mitigating personal convictions and concerns around the virus. The needs that journalism satisfies in a crisis are at odds with practices of journalism itself, but perhaps the metadiscourse provides the very antidote for how journalists best mitigate the crisis and stay anchored within the norms and ethics of the profession.

## References

[bibr1-0002764221992813] Allisop J. (2020, 5 18). The media industry’s preexisting conditions. Columbia Journalism Review. https://www.cjr.org/the_media_today/layoffs_buzzfeed_quartz_vice.php

[bibr2-0002764221992813] Bauman Z. Bordoni C. (2014). State of crisis. Polity Press.

[bibr3-0002764221992813] Benton J. (2020a, 3 18). The *Los Angeles Times* uses its physician owner to help explain the science behind the coronavirus. NiemanLab. https://www.niemanlab.org/2020/03/the-l-a-times-uses-its-physician-owner-to-help-explain-the-science-behind-the-coronavirus/

[bibr4-0002764221992813] Benton J. (2020b, 3 19). “Total annihilation”: Coronavirus may just be the end for many alt-weeklies. NiemanLab. https://www.niemanlab.org/2020/03/total-annihilation-coronavirus-may-just-be-the-end-for-many-alt-weeklies/

[bibr5-0002764221992813] Berkowitz D. (2000). Doing double duty: Paradigm repair and the Princess Diana what-a-story. Journalism, 1(2), 125-143. 10.1177/146488490000100203

[bibr6-0002764221992813] Broad G. M. Ball-Rokeach S. J. Ognyanova K. Stokes B. Picasso T. Villanueva G. (2013). Understanding communication ecologies to bridge communication research and community action. Journal of Applied Communication Research, 41(4), 325-345. 10.1080/00909882.2013.844848

[bibr7-0002764221992813] Candlin C. N. (2014). General editor’s preface. In Gunnarsson B. L. Linell P. Nordberg B. (Eds.), The construction of professional discourse (pp. viii-xiv). Routledge.

[bibr8-0002764221992813] Carlson M. (2016). Metajournalistic discourse and the meanings of journalism: Definitional control, boundary work, and legitimation. Communication Theory, 26(4), 349-368. 10.1111/comt.12088

[bibr9-0002764221992813] Carlson M. Lewis S. C. (Eds.). (2015). Boundaries of journalism: Professionalism, practices and participation. Routledge.

[bibr10-0002764221992813] Carvalho A. (2008). Media (ted) discourse and society: Rethinking the framework of critical discourse analysis. Journalism studies, 9(2), 161-177. 10.1080/14616700701848162

[bibr11-0002764221992813] Christians C. G. Glasser T. McQuail D. Nordenstreng K. White R. A. (2010). Normative theories of the media: Journalism in democratic societies. University of Illinois Press.

[bibr12-0002764221992813] Eisner J. (2020, 6 8). The uncertain future of Jewish news media. Columbia Journalism Review. https://www.cjr.org/analysis/jewish-news-media-chronicle-pandemic.php

[bibr13-0002764221992813] Fairclough N. (2001). Critical discourse analysis. In McHoul A. Rapley M. (Eds.), How to analyze talk in institutional settings: A casebook of methods (pp. 25-38). Continuum Press.

[bibr14-0002764221992813] Fürsich E. (2009). In defense of textual analysis: Restoring a challenged method for journalism and media studies. Journalism Studies, 10(2), 238-252. 10.1080/14616700802374050

[bibr15-0002764221992813] Garman A. (2019). The public sphere and journalism. Oxford Research Encyclopedia: Communication. https://oxfordre.com/communication/view/10.1093/acrefore/9780190228613.001.0001/acrefore-9780190228613-e-880

[bibr16-0002764221992813] Grau M. (2020, 3 27). Tips for covering the coronavirus from a veteran Wall Street Journal disaster reporter. Poynter. https://www.poynter.org/reporting-editing/2020/tips-for-covering-the-coronavirus-from-a-veteran-wall-street-journal-disaster-reporter/

[bibr17-0002764221992813] Gupta N. Flueckiger S. (2020, 4 6). Racism, misinformation, inclusion: How to ethically cover COVID-19. World News Publishing Focus. https://wan-ifra.org/2020/04/racism-misinformation-inclusion-how-to-ethically-cover-covid-19/

[bibr18-0002764221992813] Harris L. (2020, 5 11). “Numbers alone aren’t enough”: An interview with Caroline Chen. Columbia Journalism Review. https://www.cjr.org/q_and_a/caroline-chen-propublica-data.php

[bibr19-0002764221992813] Houston J. B. Hawthorne J. Perreault M. F. Park E. H. Goldstein Hode M. Halliwell M. R. Griffith S. A. (2015). Social media and disasters: A functional framework for social media use in disaster planning, response, and research. Disasters, 39(1), 1-22. 10.1111/disa.12092 25243593

[bibr20-0002764221992813] Katz E. (1989). Journalists as scientists. American Behavioral Scientist, 33(2), 238-246. 10.1177/0002764289033002022

[bibr21-0002764221992813] Lewis S. C. (2020). The objects and objectives of journalism research during the coronavirus pandemic and beyond. Digital Journalism, 8(5), 681-689. https://www.tandfonline.com/doi/abs/10.1080/21670811.2020.1773292

[bibr22-0002764221992813] Matloff J. (2020, 5 15). Covering anti-lockdown protests: “Time, distance, and shielding.” Columbia Journalism Review. https://www.cjr.org/analysis/covid-19-protest-journalist-safety.php

[bibr23-0002764221992813] McFarlane A. C. Norris F. H. (2006). Definitions and concepts in disaster research. In Norris F. H. Galea S. Friedman M. J. Watson P. J. (Eds.), Methods for disaster mental health research (pp. 3-19). Guilford Press.

[bibr24-0002764221992813] Nelson J. (2020, 3 27). Coronavirus: News media sounded the alarm for months but few listened. Editor & Publisher. https://www.editorandpublisher.com/stories/coronavirus-news-media-sounded-the-alarm-for-months-but-few-listened,1291?

[bibr25-0002764221992813] Paulussen S. Harder R. A. (2014). Social media references in newspapers: Facebook, Twitter and YouTube as sources in newspaper journalism. Journalism Practice, 8(5), 542-551. 10.1080/17512786.2014.894327

[bibr26-0002764221992813] Pavlik J. V. (2013). Innovation and the future of journalism. Digital Journalism, 1(2), 181-193. 10.1080/21670811.2012.756666

[bibr27-0002764221992813] Perreault G. Bell T. R. (2020). Towards a “digital” sports journalism: Field theory, changing boundaries and evolving technologies. Communication & Sport. Advance online publication. 10.1177/2167479520979958

[bibr28-0002764221992813] Perreault G. Johnson B. Klein L. (2020). Covering hate: Field theory and journalistic role conception in reporting on White nationalist rallies. Journalism Practice, 1-17. 10.1080/17512786.2020.1835525

[bibr29-0002764221992813] Perreault G. Stanfield K. Luttman S. (2020). “Why the h** l is there a White House correspondents’ dinner?” Boundary work in political journalism. Journalism Practice, 14(9), 1142-1158. 10.1080/17512786.2019.1685901

[bibr30-0002764221992813] Perreault G. Vos T. (2020). Metajournalistic discourse on the rise of gaming journalism. New Media & Society, 22(1), 159-176. 10.1177/1461444819858695

[bibr31-0002764221992813] Perreault M. F. (2020, 8 9). Journalism beyond the command post [Paper presentation]. Association of Education in Journalism and Mass Communication Annual Conference San Francisco, California.

[bibr32-0002764221992813] Perreault M. F. Houston J. B. Wilkins L. (2014). Does scary matter? Testing the effectiveness of new National Weather Service tornado warning messages. Communication Studies, 65(5), 484-499. 10.1016/j.ijdrr.2016.04.006

[bibr33-0002764221992813] Schudson M. (1982). The politics of narrative form: The emergence of news conventions in print and television. Daedalus, 111(4), 97-112. https://www.jstor.org/stable/20024819

[bibr34-0002764221992813] Soon-Shiong P. (2020, 3 17). The science behind the cornavirus. Los Angeles Times. https://www.latimes.com/science/science-behind-coronavirus-123

[bibr35-0002764221992813] Spialek M. L. Czlapinski H. M. Houston J. B. (2016). Disaster communication ecology and community resilience perceptions following the 2013 central Illinois tornadoes. International Journal of Disaster Risk Reduction, 17(August), 154-160. 10.1016/j.ijdrr.2016.04.006

[bibr36-0002764221992813] Stephens M. (1989). A history of news. Oxford University Press.

[bibr37-0002764221992813] Trewinnard T. (2020, 4 13). The coronavirus crisis will eventually end, but the distributed newsroom is here to stay. NiemanLab. https://www.niemanlab.org/2020/04/the-coronavirus-crisis-will-eventually-end-but-the-distributed-newsroom-is-here-to-stay/

[bibr38-0002764221992813] Veil S. Reynolds B. Sellnow T. L. Seeger M. W. (2008). CERC as a theoretical framework for research and practice. Health Promotion Practice, 9(4 Suppl.), 26S-34S. 10.1177/1524839908322113 18936257

[bibr39-0002764221992813] Vos T. P. (2016). Journalistic fields. In Witschge T. Anderson C. W. Domingo D. Hermida A. (Eds.), The Sage handbook of digital journalism (pp. 383-396). Sage 10.4135/9781473957909.n26

[bibr40-0002764221992813] Vos T. P. Perreault G. P. (2020). The discursive construction of the gamification of journalism. Convergence, 26(3), 470–485. 10.1177/1354856520909542

[bibr41-0002764221992813] Wahl-Jorgensen K. (2020). “An emotional turn in journalism studies?” Digital Journalism, 8(2), 175-194. 10.1080/21670811.2019.1697626

[bibr42-0002764221992813] Weaver D. H. Willnat L. (2016). Changes in US journalism: How do journalists think about social media? Journalism Practice, 10(7), 844-855. 10.1080/17512786.2016.1171162

[bibr43-0002764221992813] Welton C. (2020, 4 2). In a moment of uncertainty, TIME is certain of its 97-year mission. Folio. https://www.foliomag.com/time-certain-of-its-mission/

[bibr44-0002764221992813] Zelizer B. (2015). Terms of choice: Uncertainty, journalism, and crisis. Journal of Communication, 65(5), 888-908. 10.1111/jcom.12157

